# Supplementary material for: Identification of photoperiod-induced specific miRNAs in the adrenal glands of Sunite sheep (Ovis aries)
Source: Front Vet Sci. 2022 Jul 22;9:888207. doi: 10.3389/fvets.2022.888207 (PMC9354845; doi:10.3389/fvets.2022.888207)
Supplement: Supplementary file 1 [file Data_Sheet_1.ZIP › Supplementary file/miRDeep2 file.zip!/miRDeep2 file/Novel_Structure/SP42A3/Novel_296.pdf]

Provisional ID	:	NC_019459.2_3737
Score total	:	26.1
Score for star read(s)	:	3.9
Score for read counts	:	19
Score for mfe	:	1.6
Score for randfold	:	1.6
Score for cons. seed	:	
Total read count	:	49
Mature read count	:	48
Loop read count	:	0
Star read count	:	1

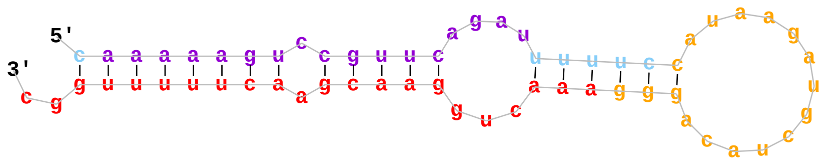

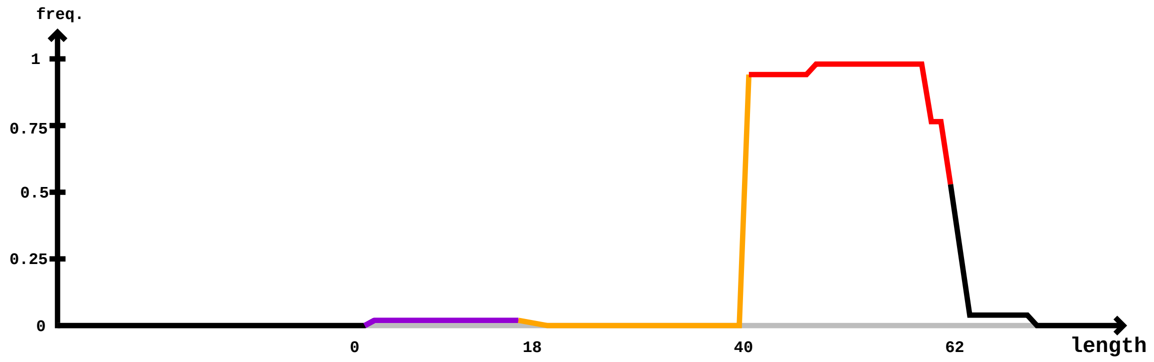


```
                                 Star                                   Mature
5'- ugcacgugugugucc guauauuggguugaacaaaaaguccguucagauuuuuccauaagaugcuacagggaaacuggaacgaacuuuuuggccaacccaauaauaauca  -3'   obs
    ugcacgugugugucc guauauuggguugaacaaaaaguccguucagauuuuuccauaagaugcuacagggaaacuggaacgaacuuuuuggccaacccaauaauaauca        exp
    .((((....)))).....((((((((((..((((((((.(((((....((((((.............))))))...))))).))))))))..)))))))))).......   reads   mm   sample
    ...............................aaaaaguccguucagau.............................................................   1       0    seq
    ......................................................................aaacuggaacgaacuuuuu....................   11      0    seq
    ......................................................................aaacuggaacgaacuuuuugg..................   12      0    seq
    ......................................................................aaacuggaacgaacuuuuuggc.................   23      0    seq
    ......................................................................aaacuggaaUgaacuuuuuggc.................   1       1    seq
    ......................................................................aaacuggaacgaacuuuuuggcc................   1       0    seq
    .............................................................................aacgaacuuuuuggccaacccaa.........   2       0    seq
```
